# Bioinspired High-Performance Bilayer, pH-Responsive Hydrogel with Superior Adhesive Property

**DOI:** 10.3390/polym14204425

**Published:** 2022-10-19

**Authors:** Shulan Jiang, Li Xia

**Affiliations:** 1 School of Mechanical Engineering and Electronic Information, China University of Geosciences (Wuhan), Wuhan 430074, China; 2 Tribology Research Institute, School of Mechanical Engineering, Southwest Jiaotong University, Chengdu 610031, China

**Keywords:** bilayer hydrogel, soft actuator, pH-responsive, bending, adhesive property

## Abstract

Soft actuators have attracted extensive attention for promising applications in drug delivery, microfluidic switches, artificial muscles and flexible sensors. However, the performance of pH-responsive hydrogel actuators, such as regarding reversible bending property and adhesive property, remains to be improved. In this study, inspired by drosera leaves, we have fabricated high-performance bilayer, pH-responsive poly(acrylamide-acrylic acid-3-acrylamidophenylboronic acid)(P(AAm-AAc-3-AAPBA)) based on the copolymers of AAm, AAc and 3-AAPBA. The pH-sensitive actuators were fabricated by ultraviolet polymerization of the P(AAm-AAc-3-AAPBA) layer as the active actuating layer and the PAAm layer as the auxiliary actuating layer. The effects of pH, glucose concentration and content of 3-AAPBA on bending behavior of P(AAm-AAc-3-AAPBA)/PAAm bilayer actuators were discussed. By tuning the pH of media, the soft actuator could achieve fast and large-amplitude bidirectional bending behaviors. The bending orientation and bending degree can be reversibly and precisely adjusted. More importantly, P(AAm-AAc-3-AAPBA) hydrogel shows good adhesive property in polyvinyl alcohol (PVA) solution; thus, complex structures have been fabricated. In addition, the bilayer hydrogel structures have been demonstrated as soft actuators, bionic flowers and bionic manipulators. The proposed pH-responsive bilayer actuator shows great potential for drug delivery and other medical systems.

## 1. Introduction

Among various materials, stimuli-responsive hydrogels are widely used in many fields, such as drug delivery, microfluidic switches, artificial muscles, flexible sensors, etc. [1,2]. They have the ability to generate reversible shape deformation, color change or variation in mechanical properties upon introduction of environmental stimuli, including temperature, pH, light and certain chemical triggers [3]. Further, pH-responsive hydrogels are an important class of smart materials, where pH, as an external stimulus, is an important trigger to change the sol–gel transition, surface activity, chain conformation, swelling capacity, drug delivery kinetics, fluorescence behavior, etc. [4,5]. Their unique properties result in great interest regarding various applications, such as drug delivery, gene delivery, sensors, surfaces, membranes and chromatography. Swelling capacity is one of the most fundamental and important characteristics, which can dictate stability, shape and volume changes in hydrogels [6]. The stimulus response deformation of hydrogels includes expansion/contraction and bending/straightening. The bending/straightening motion of the hydrogel depends on parameters such as shape and size. Hydrogel actuators are based on design of anisotropic structures driven by swelling/de-swelling of polymer hydrogels for changing shape [5]. Recently, pH responsiveness has attracted attention, and many reports have been published on fabrication of pH-responsive smart hydrogels. For example, Ma et al. [7] reported a pH-responsive and thermo-responsive bilayer hydrogel soft actuator, combining a collapsed thermo-responsive graphene oxide-poly(*N*-isopropylacrylamide) hydrogel layer with a pH-responsive perylene-bisimide-functionalized hyperbranched polyethylenimine hydrogel layer via macroscopic supramolecular assembly. Wu et al. [8] proposed a novel hydrogel actuator with pH-responsive fluorescence and thermally induced actuating behavior. Bi et al. [9] explored smart bilayer polyacrylamide/DNA hydrogel film actuators with programmable pH-responsive properties directed by sequences of DNA crosslinking units undergoing reversible and large-amplitude macroscopic shape transformations. Hong et al. [10] reported that a boronic-acid-tethered alginate polymer satisfies the properties for hydrogel attachment and assembly. Many studies on hydrogel actuators were inspired by the natural actuating system in plants. For example, the glands of drosera can secrete mucus to attract and trap insects. Kong et al. [11] reported that temperature-responsive dual-actuators learn from movement of tentacles through synergy of the double-layer structure (shrinking in the adaxial half of the tentacle and swelling in the abaxial half). Han et al. [5] reported a pH-responsive hydrogel actuator for lipophilic drug delivery with polyacrylic acid and polyacrylamide hydrogels. Further, 3-AAPBA is an important monomer that is sensitive to glucose. It has been widely used in glucose sensor and other glucose-sensitive hydrogels [12]. 3-AAPBA is also sensitive to the pH of the environment. The volume of 3-AAPBA will change with pH. When the pH is high, it will be saturated in ionized state; when the pH is low, it will be hydrophobic in non-ionized state. AAc is also widely used for pH-sensitive hydrogels [13]. It was found that the copolymers of 3-AAPBA and AAc have not been used in pH-sensitive soft actuators with a large bending angle, fast response speed and superior adhesive property.

To improve the performance of pH-responsive hydrogel actuators, such as reversible bending property and adhesive property, we have fabricated a pH-sensitive hydrogel of P(AAm-AAc-3-AAPBA) based on the copolymers of AAm, 3-AAPBA and AAc. Then, pH-sensitive actuators were fabricated by UV polymerization of the P(AAm-AAc-3-AAPBA) layer as the active actuating layer and the PAAm layer as the auxiliary actuating layer, which were inspired by the smart drosera system. The effects of pH, glucose concentration and content of 3-AAPBA on the bending behavior of the P(AAm-AAc-3-AAPBA)/PAAm bilayer were discussed, and the reversibility of the bilayer structures and the bonding property were investigated. The bilayer structures were demonstrated as grasper, flower- and hand-like actuators.

## 2. Materials and Methods

### 2.1. Materials

Diethoxyacetophenone (DEAP, 99%) and 3-acrylamidophenylboronic acid (3-AAPBA, 99%) were purchased from Aladdin Co. Ltd (Shanghai, China). Acrylamide (AAm, 99%) was purchased from Maclin (Shanghai, China). Acrylic acid (AAc, 99%) was purchased from Jindong Tianzheng Fine Chemical Reagent Factory (Tianjin, China). *N,N’*-Methylene-bis (acrylamide) (BIS, 99%) was purchased from Adamas-beta, Shanghai China. Dimethylsulfoxide (DMSO, 99%) was obtained from Chengdu Jinshan Chemical Reagent Company (Chengdu, China). Polyvinyl alcohol (PVA, 99%), NaOH (AR) and HCl (AR) were purchased from Chengdu Kelong Chemical Reagent Company (Chengdu, China). All the reagents were used as received.

### 2.2. Synthesis of the Bilayer Hydrogels

**Figure 1:**
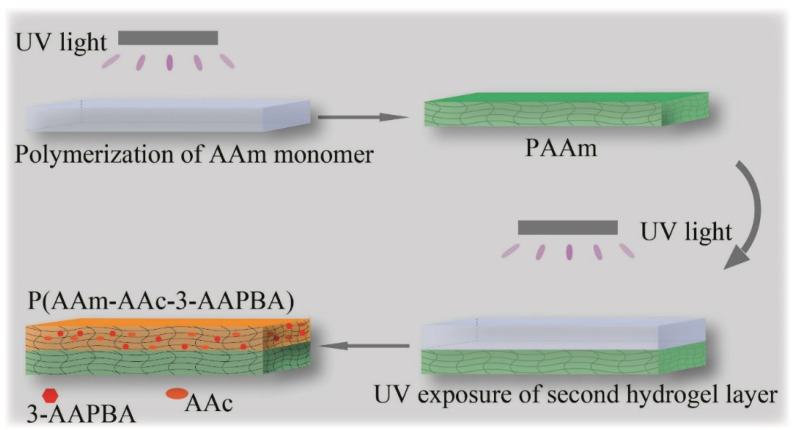
The fabrication schematics of the pH-responsive bilayer structure.

The bilayer hydrogel was prepared by two-step UV-polymerization, which is facile to manipulate. First, 1.05 g AAm, 0.03 g BIS, 3 g DMSO and 0.3 g of DEAP were mixed with 6 mL deionized (DI) water, yielding a pre-monomer solution of PAAm. Further, 3-AAPBA was first mixed with 3 g DMSO by ultrasonic vibration, and 0.6 g AAm, 300 μL AAc, 0.03 g BIS and 0.3 g of DEAP were added into the above solution and then mixed with 6mL DI water to obtain 2.5 wt% 3-AAPBA pre-monomer solution of P(AAm-AAc-3-AAPBA) [14]. The two pre-monomer solutions were placed in a vacuum reactor and stirred for 15 min, respectively. The preparation process is shown in Figure 1. At first, PAAm pre-monomer solution was transferred into a sandwich glass mold with the solution height of 1 mm exposed to UV light (λ = 365 nm) for 5 min at room temperature. Then, P(AAm-AAc-3-AAPBA) pre-monomer solution was injected into the above glass mold on the PAAm layer with the solution height of 1mm and solidified by UV exposure for 6 min at room temperature. The bilayer hydrogel structures (60 mm × 20 mm × 2 mm) were obtained. It was washed by DI water three times to remove residual monomers. The penetration of P(AAm-AAc-3-AAPBA) monomer into the surface of PAAm layer will form an interpenetration network, which connects two layers tightly. The bilayer structures were cut into a strip of 20 mm × 2 mm × 2 mm or designed shape.

### 2.3. Characterization

Scanning electron microscopy (SEM, Phenom Pro, Phenom World, Eindhoven, The Netherlands) was used to characterize the interface morphology of the sample. The bilayer hydrogel was quickly frozen and cut under liquid nitrogen. Then, it was freeze-dried by freezer dryer for 24 h. The chemical elements of the samples were characterized by Fourier transform infrared spectroscopy (FTIR, Thermo Scientific Nicolet iS5, Waltham, MA, USA). Different types of bilayer hydrogel structures can be fabricated with AAm, AAc and 3-AAPBA. To select the most promising one for further studies, we have fabricated and tested four types of bilayer structures, including P(AAm-AAc-3-AAPBA)/P(AAm-AAc) (type 1) bilayer hydrogel, P(AAm-3-AAPBA)/P(AAm-AAc) bilayer hydrogel (type 2), P(AAm-3-AAPBA)/PAAm bilayer hydrogel (type 3) and P(AAm-AAc-3-AAPBA)/PAAm (type 4). The pristine structure and bending behaviors of the bilayer structures and soft actuators were recorded by a digital camera (Canon FS100A, Tokyo, Japan) at different times. The bending angles of the structures were tested by image J.

## 3. Results and Discussion

**Figure 2:**
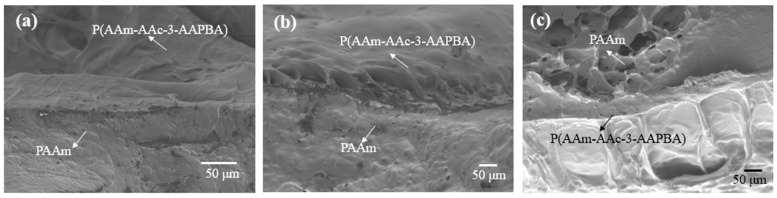
(**a**–**c**) SEM images of the bilayer structures that were treated in acidic environment (with a pH of 1), DI water and alkaline environment (at pH 13), respectively.

We have prepared three samples, which were placed in acid (with a pH of 1), neutral (DIW) and alkaline (with the pH of 13) solution for 30 min to realize swelling equilibrium state, and then they were freeze-dried for 24 h after freezing and breaking in liquid nitrogen. The SEM images of the three samples with different states are shown in Figure 2. The internal structure of the P(AAm-AAc-3-AAPBA) layer is very compact (Figure 2a) due to discharge of a large amount of water and formation of internal hydrogen bonds. When the sample was treated in DI water (with a pH larger than the pKa 4.7 of AAc) [14], carboxylic acid groups of AAc were ionized and the –COO– groups were formed. Therefore, the hydrophilicity of the P(AAm-AAc-3-AAPBA) layer increased. Meanwhile, the water absorption capacity of the bilayer hydrogel increases with pH. Therefore, some internal pores emerge, as shown in Figure 2b. For the samples treated in alkaline solution (Figure 2c), massive borate anions and acrylate ions were formed, so the hydrogel absorbed water and swelled. The cross-section of the hydrogel is obviously loose and porous, as shown in Figure 2c. In addition, the SEM images all show a clear boundary of the bilayer hydrogels, which proves a close connection of the two layers of the hydrogels.

**Figure 3:**
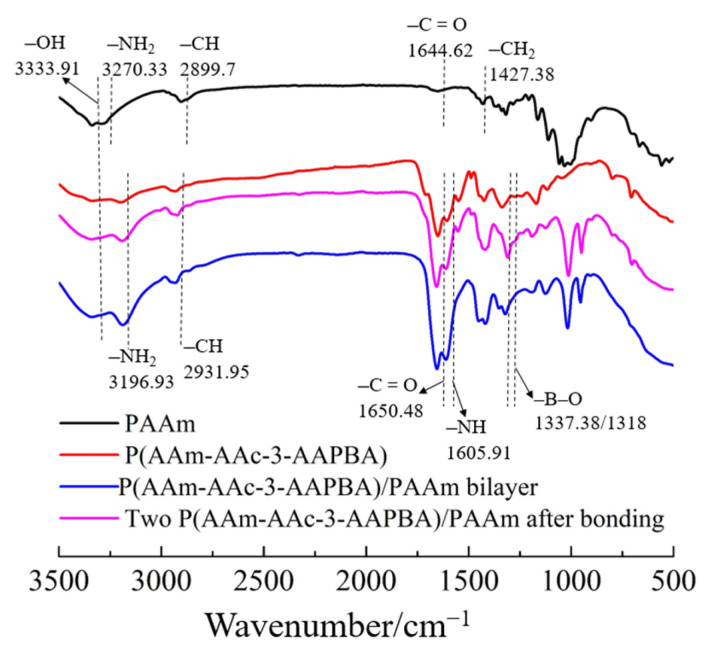
FTIR spectra of PAAm hydrogel, P(AAm-AAc-3-AAPBA) hydrogel, P(AAm-AAc-3-AAPBA)/PAAm bilayer hydrogel and two P(AAm-AAc-3-AAPBA)/PAAm bilayer hydrogel after bonding in PVA solution, respectively.

The structure of PAAm hydrogel, P(AAm-AAc-3-AAPBA) hydrogel, P(AAm-AAc-3-AAPBA)/PAAm bilayer before bonding in PVA solution and two P(AAm-AAc-3-AAPBA)/PAAm bilayers after bonding in PVA solution were characterized by FTIR. The spectra are shown in Figure 3. The FTIR curve of PAAm shows the presence of –C=O (1644.62 cm^−1^), –NH_2_ (3270.33 cm^−1^), –CH (2899.7 cm^−1^) and –CH_2_ (1427.38 cm^−1^) groups, which proves the formation of a PAAm layer. The FTIR spectrum of P(AAm-AAc-3-AAPBA) shows the presence of amide I group –C=O (1650.48 cm^−1^) and amide II group –NH_2_ (3196.93 cm^−1^). The presence of –CH (2931.95 cm^−1^), –NH (1605.91 cm^−1^) and –B–O (1337.38 cm^−1^) groups indicates polymerization of AAm, AAc and 3-AAPBA. In addition, the –C=O group in the FTIR spectrum of P(AAm-AAc-3-AAPBA) has a shift compared with that of PAAm, which indicates formation of hydrogen bonds. The characteristic peaks of P(AAm-AAc-3-AAPBA)/PAAm are almost the same as those of two P(AAm-AAc-3-AAPBA)/PAAm after bonding.

**Figure 4:**
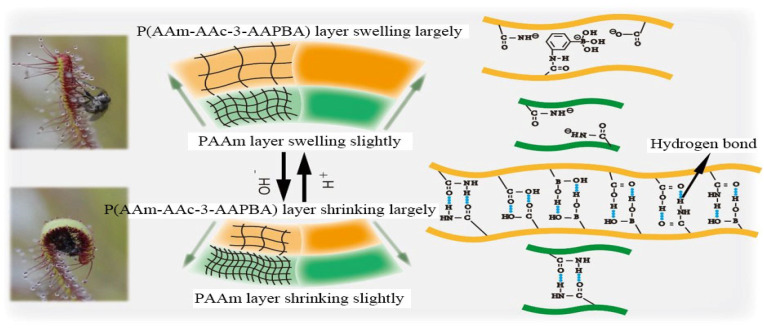
Optical images of the drosera and bending mechanism of the bilayer structures.

The working mechanism of the P(AAm-AAc-3-AAPBA)/PAAm bilayer structures is shown in Figure 4. The pictures of drosera, which is attracting and catching insects by sweet and sticky mucilage secreted by glandular tentacles on leaves, respectively, are shown in the inset of Figure 4. The bilayer hydrogel soft actuator produces positive bending deformation under extremely acidic conditions. As the pH of the solution approaches neutral gradually, the bilayer hydrogel produces positive bending at first, and then produces reverse bending deformation. As the solution becomes alkalinous, the bilayer hydrogel bends reversely. When the pH of the solution is sufficiently low, formation of hydrogen bonds between AAm, AAc and 3-AAPBA of the P(AAm-AAc-3-AAPBA) hydrogel and the hydrophobic protonated states of AAm, AAc and 3-AAPBA contributes to the compact internal structures of P(AAm-AAc-3-AAPBA). Thus, the bilayer hydrogel structures bend positively. With an increase in solution pH, the bilayer hydrogel produces reverse bending deformation. AAc and 3-AAPBA ionize to form acrylate ions and borate ions, and the internal hydrogen bonds are broken. The water absorbing ability of P(AAm-AAc-3-AAPBA) becomes larger. Only part of NH_2_ ionized in the PAAm hydrogel layer. Therefore, when the water absorbing ability of P(AAm-AAc-3-AAPBA) is larger than that of the PAAm layer, the bilayer hydrogel structure bends reversely [15,16,17]. The volume change in the PAAm hydrogel layer is positively correlated with that of the P(AAm-AAc-3-AAPBA) layer, but the variation is much smaller than that of P(AAm-AAc-3-AAPBA) [18]. Simultaneously, the hydrogels of AAc and AAm have good water absorption ability and softness, so the bi-directional bending angle can surpass 360°.

The AAc in the P(AAm-AAc-3-AAPBA) layer is pH-responsive, with a pKa value of 4.25 [19]. The carboxylic acid group in AAc will be ionized when pH > 4.25. The hydrogels absorb water and the volume increases because of the increment of carboxylate anions [19]. The bilayer structures with different composition types have different bending states. We have studied the four composition types of bilayer hydrogels, namely P(AAm-AAc-3-AAPBA)/P(AAm-AAc) (type 1) bilayer hydrogel, P(AAm-3-AAPBA)/P(AAm-AAc) bilayer hydrogel (type 2), P(AAm-3-AAPBA)/PAAm bilayer hydrogel (type 3) and P(AAm-AAc-3-AAPBA)/PAAm (type 4). We have tested the bending performance of the four types. Figure S1 (see Supplementary Materials) shows that the P(AAm-AAc-3-AAPBA)/PAAm bilayer structures exhibit large bending angles in an acidic environment (with a pH of 1). The P(AAm-AAc-3-AAPBA)/PAAm bilayer structures also show large bending angles in DI water. For the alkaline environment, P(AAm-3-AAPBA)/P(AAm-AAc) and P(AAm-AAc-3-AAPBA)/PAAm bilayer structures all exhibit good bending performance. AAc is good for the bending behavior of bilayer hydrogel soft actuator, which is contributed by different ionization properties in various pH environments. We selected the P(AAm-AAc-3-AAPBA)/PAAm bilayer structures, which exhibit good bending behavior for the following experiments.

The 3-AAPBA hydrogel is pH-responsive (the pKa ranges from 8.2 to 8.6) [20]. When the pH of the environment is larger than the pKa of the 3-AAPBA hydrogel, it will be ionized and the boronic acid radical ions will form. Therefore, the Donnan osmotic pressure will increase, thus resulting in an increment of water absorbing ability. However, when the pH of the environment is lower than the pKa, the 3-AAPBA hydrogel maintains hydrophobic protonation states. The effect of concentration of 3-AAPBA on bending behavior of the bilayer structures was studied. We have tested the bending properties of the bilayer hydrogel structures with different concentrations of 3-AAPBA in different pH environments (0, 2.5, 5, 7.5 and 10 wt%). The results showed the bilayer hydrogel structures with the concentrations of 2.5 and 7.5 wt% had quick bending speed and large bending angles (Figure S2, see Supplementary Materials). We selected the concentrations of 2.5 wt% of 3-AAPBA to fabricate bilayer hydrogels in the following study.

**Figure 5:**
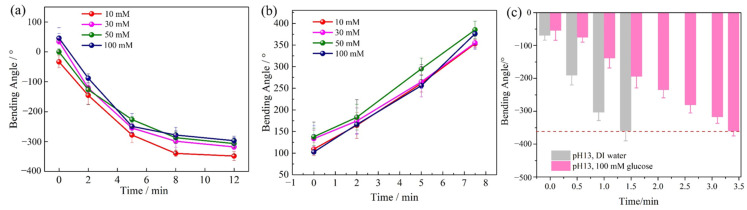
(**a**) The influence of glucose concentration on bending angle and bending rate of bilayer hydrogel structure in alkaline environment of pH13. (**b**) The influence of glucose concentration on bending angle and rate of bilayer hydrogel structure in DI water. (**c**) Comparison of bending behavior of bilayer hydrogel structure in DI water with pH13 and 100 mM glucose solution with pH13, respectively.

The bending behaviors of the bilayer hydrogel structures were tested in 10 mM, 30 mM, 50 mM and 100 mM glucose solutions with a pH of 13. It shows that the bending angles of the bilayer hydrogel structures in 50 mM and 100 mM glucose solutions are slightly smaller than those in 10 mM and 30 mM glucose solutions (Figure 5a). The bending angle of the bilayer hydrogel soft actuator in low glucose concentration is larger than that in high glucose concentration. It is supposed that the high glucose concentration in aqueous solution partially hinders water absorption of the P(AAm-AAc-3-AAPBA) hydrogel structures. Therefore, the reverse bending angle of the bilayer hydrogel tends to decrease with the rise in glucose concentration. The bending behavior of the bilayer hydrogel actuators in DI water is also tested with different glucose concentrations. The results are shown in Figure 5b. The bending angle and bending rate show irregular subtle changes with different glucose concentrations. To better show the effect of glucose on the bending property of the bilayer hydrogel structures, we tested the bilayer hydrogel structures in an alkaline environment with and without glucose. As shown in Figure 5c, the bilayer hydrogel soft actuators bend to 360° after 1.5 min in solution with a pH of 13, while the hydrogel actuators bend to 360° after 3.3 min in glucose solution with a concentration of 100 mM and pH of 13. Therefore, the presence of glucose in an alkaline environment slows down the bending rate of the bilayer hydrogel. For a bilayer hydrogel, it is key to speed up the bending rate and increase the bending angle. In the following study, the property of the bilayer hydrogel actuator responding to different pH values will be discussed.

**Figure 6:**
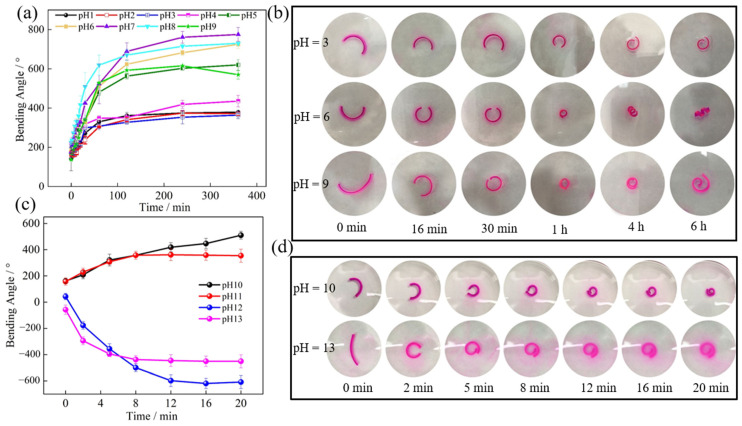
(**a**) The relationship between bending angle and time for bilayer hydrogel soft actuators placed in solution with a pH of 1–9 within 6 h. (**b**) Photographs of bilayer hydrogel structures in solution with a pH of 3, 6 and 9, respectively. (**c**) The relationship between bending angle and time for bilayer hydrogel actuators placed in solution with a pH of 10–13 within 20 min. (**d**) Photographs of bilayer hydrogel structures placed in solution with a pH of 10 and 13, respectively.

It was reported that the 3-AAPBA is in an incomplete ionization state in the pH range of 7–9.5 [20]. Therefore, it is very important to study the bending behavior of P(AAm-AAc-3-AAPBA)/PAAm bilayer structures in different pH environments. We have observed that the bilayer structure bends to the P(AAm-AAc-3-AAPBA) layer and then bends to the PAAm layer in a certain pH range in the experiment. Therefore, we studied the pH range of 1–9 and 10–13 separately. As shown in Figure 6a,b, the bilayer soft actuator bends to the P(AAm-AAc-3-AAPBA) layer and the bending angle is similar in the pH range of 1–3. The unidirectional bending angle and rate of the hydrogel structure show an increasing tendency at a pH of 4 and 5. However, the bilayer hydrogel soft actuator bends to the P(AAm-AAc-3-AAPBA) layer at the beginning and then reverses within 6 h in the solution at pH 6–9. It is probably because the ionicity of AAc and 3-AAPBA increase with pH, so the water absorbing ability increases. As shown in Figure 6c,d, the bilayer soft actuators placed in the solutions of pH 10 and pH 11 have positive bending deformation within 20 min, and the bending deformation angle of a sample placed in a solution of pH 10 is greater with prolongation of time. The hydrogel actuator produces reverse bending deformation rapidly in a solution environment of pH 12 and pH 13, as shown in Figure 6c. The bending rate and bending angle of the bilayer hydrogel in the solution of pH 13 is greater than in pH 12 at an early stage. Then, the bending rate and angle of the structure placed in the solution of pH 12 gradually surpass those of the structure placed in the solution of pH 13. It is supposed that the improvement in –NH_2_ ionization of PAAm hydrogel when placed in pH 13 increases the water absorption of PAAm hydrogel and thus hinders the reverse bending behavior. The relationship between maximum bending angle and pH is shown in Table 1. At pH 7, the maximum bending angle of the bilayer structure is 775 ± 35.36°, while the deformation time is 360 min. The maximum bending angle of the bilayer structure is −608 ± 2.77° after being placed in pH 12 for 20 min. In total, the response speed of the bilayer structures is faster in acidic solution than in alkaline solution, while the bending angle is smaller in acidic solution than in alkaline solution.

**Figure 7:**
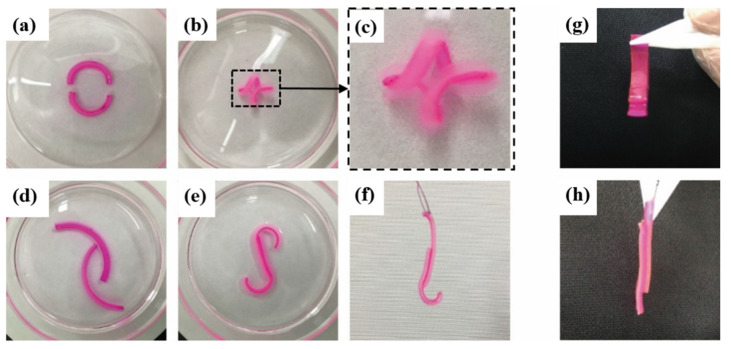
Bonding different parts of P(AAm-AAc-3-AAPBA) layer in two bilayer hydrogel structures: (**a**–**c**) two ends, (**d**–**f**) middle parts, (**g**–**h**) surface of two P(AAm-AAc-3-AAPBA) layers to form different patterns. The size of the hydrogel strip is 20 mm × 2 mm × 2 mm in (**a**–**c**) and (**d**–**f**), while the size of the hydrogel strip in (**g**–**h**) is 20 mm × 3 mm × 2 mm.

Except for bending behaviors, it is found that the PAAm/P(AAm-AAc-3-AAPBA) bilayer hydrogel shows superior adhesion property when bonding with P(AAm-AAc-3-AAPBA)/PAAm in acid PVA solution after 20 min, as shown in Figure 7. The bonding patterns of the P(AAPBA-AAm-AAc)/PAAm bilayer hydrogels are shown in Figure 7. Two P(AAm-AAc-3-AAPBA)/PAAm bilayers could bond together by dynamic hydrogen bonds generated by the boronic acid groups in 3-AAPBA, amino-groups in AAm and carboxylic acid groups in AAc in acidic PVA medium [21,22]. Moreover, the carboxylic acid groups in AAc and boronic acid groups in 3-AAPBA and partially amino-groups will be ionized in higher pH solution, causing the increment and swelling of internal osmotic pressure of the hydrogels, respectively. Therefore, the P(AAm-AAc-3-AAPBA)/PAAm bilayer structures exhibit good bending behavior and bonding property simultaneously. As shown in Figure 7, various bonding patterns have been demonstrated that were bonded with different parts, such as two ends, middle parts and surface of P(AAm-AAc-3-AAPBA) layer, respectively. The patterns exhibit relatively stable properties.

**Figure 8:**
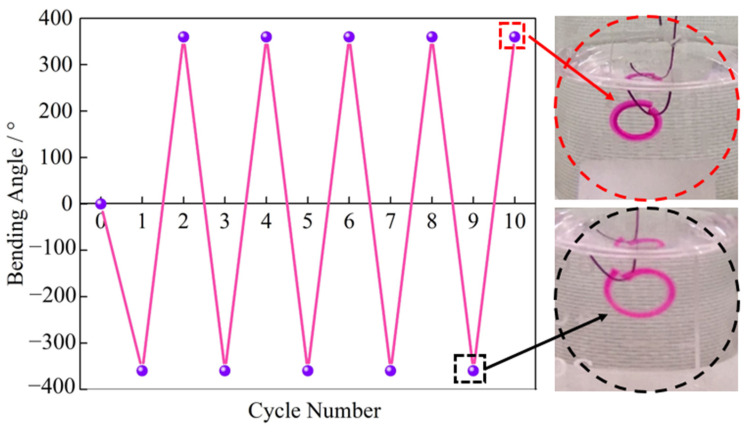
Reversible bending behaviors and the enlarged images of the bilayer structures bending at pH 1 (360°) and 13 (−360°). The size of the hydrogel strip is 20 mm × 2 mm × 2 mm.

Repeatedly and reversibly bending is of great importance in real applications. The reversible bending performance was evaluated in this study. One end of the bilayer hydrogel strip was fixed with a thin iron wire. It was first placed in an alkaline solution at pH 13. When it bended to about 360°, it was transferred to an acidic solution at pH 1. This process was repeated five times. The results are shown in Figure 8. The bilayer structures exhibit excellent pH reversibility. It was observed that the bilayer hydrogel showed structural stability without damage even after cycling tests.

**Figure 9:**
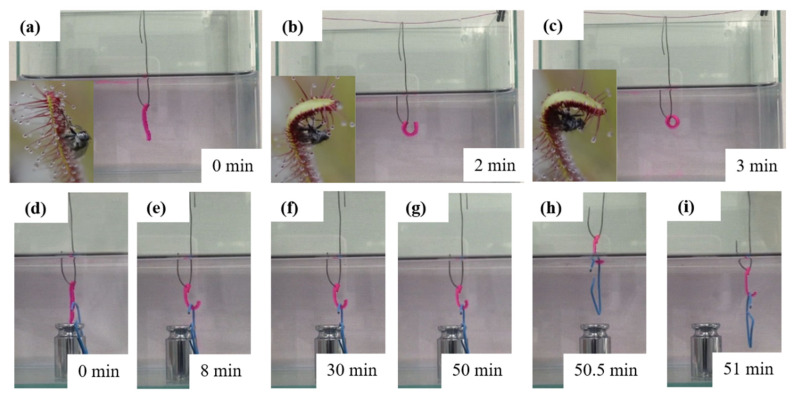
(**a**–**c**) The bending behavior of drosera-inspired bilayer soft actuator. (**d**–**g**) The process of soft actuator grabbing a clip. (**h**–**i**) The soft actuator pulling out the clip and putting in water, respectively. The size of the hydrogel strip is 20 mm × 2 mm × 2 mm.

The P(AAm-AAc-3-AAPBA)/PAAm bilayer hydrogel was demonstrated as soft actuators. It precipitates water in an acidic environment, leading to large crosslinking density. When placed in alkaline solution, the bilayer hydrogel structures absorb water and the crosslinking density decreases with the increment of ionization. Crosslinking density is related to loading capacity. Therefore, the drosera-bioinspired soft actuator was fabricated in an acidic environment. As shown in Figure 9, the soft actuator bends to the P(AAm-AAc-3-AAPBA) layer gradually. The bending angle is about 320° after 2 min. It is capable of grabbing a clip (with the weight of about 1 g) and moving it freely after 50 min. The actuator can even grab the clip into the air and release the clip by placing the actuator in the alkaline solution due to the reverse bending.

**Figure 10:**
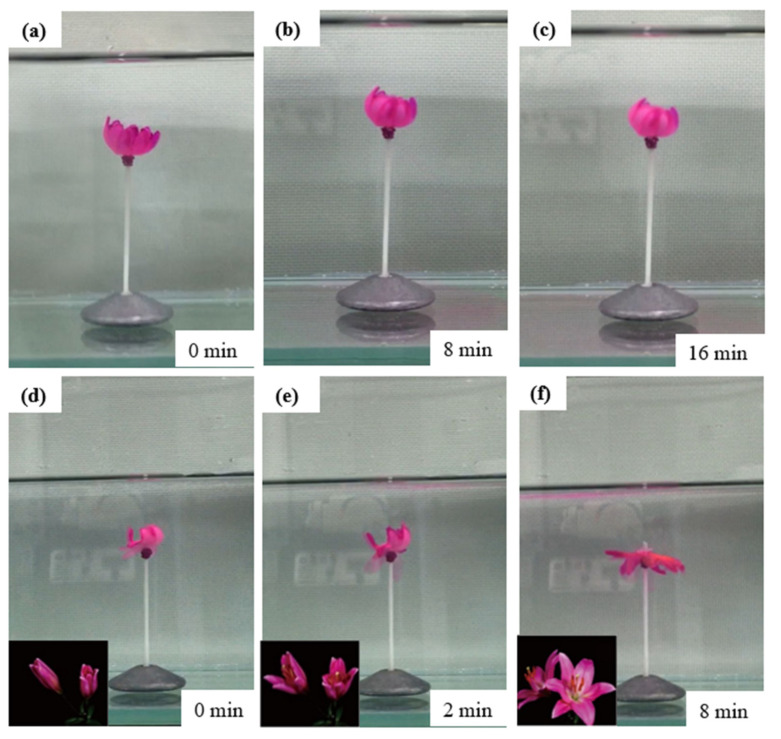
(**a**–**c**) The process of flower-like soft actuator in acidic solution. (**d**–**f**) The blooming process of the flower-like soft actuator in alkaline solution. The insets are flowers opening in nature. The maximum diameter of the bionic flower is 50 mm, and the thickness is 2 mm.

We have demonstrated the pH-responsive P(AAm-AAc-3-AAPBA)/PAAm bilayer structures as flower-shaped hydrogel soft actuator. By means of the bending property of the bilayer hydrogel, the flower-like actuator imitates the closing and opening processes of flowers in nature. As shown in Figure 10, the flower-shaped soft actuator was first placed in acidic solution. It slowly closes to form a flower bud (shown in Figure 10a–c) because the P(AAm-AAc-3-AAPBA) layer of the petals is hydrophobic. Then, the hydrogel flower bud was placed in alkaline solution; the hydrogel flower was blooming due to large water swelling capacity of the P(AAm-AAc-3-AAPBA) layer after ionization of boric acid groups and amino groups and breaking of a large number of hydrogen bonds (shown in Figure 10d–f).

**Figure 11:**
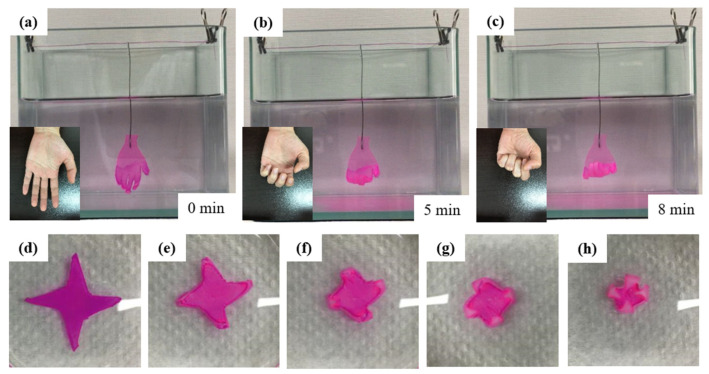
(**a**–**c**) Hand-shaped hydrogel mimicking hand grasping a fist; the width of the bionic hand is 20 mm and the thickness is 2 mm. (**d**–**h**) The deformation process of the hydrogel windmill. The maximum diameter of the imitated windmill is 20 mm, and the thickness is 2 mm.

The P(AAm-AAc-3-AAPBA)/PAAm bilayer hydrogel structure has also been fabricated as a hand-type soft actuator, as shown in Figure 11a–c. The palm part is prepared by PAAm hydrogel layer. The hand-shaped hydrogel can produce bending and deformation behavior like a hand grasping a fist in acidic solution environment. The bilayer structure is also used to prepare windmill (shown in Figure 11d–h). The volume of the hydrogel windmill decreases and the leaves are slowly rolled up to form a windmill-like hydrogel in a solution at pH 1. The bilayer hydrogel structure shows fast deformation speed and a large bending angle, so it has great potential in soft actuators.

The proposed pH-responsive bilayer hydrogel structures of P(AAm-AAC-3-AAPBA)/PAAm are fabricated by a mixing process, injecting into the glass mold and UV light irradiation for each layer separately. The bilayer structure is composed of the copolymers of AAm, AAc and 3-AAPBA, where the copolymers have not been used as bilayer structures before. We have discussed the effects of pH, glucose concentration and content of 3-AAPBA on the bending behavior of P(AAm-AAc-3-AAPBA)/PAAm bilayer structures. Through tuning the pH of media, the soft actuator could achieve a fast and large-amplitude bending angle at a relatively high pH of 10 to 13. However, at a pH of 1 to 9, the bilayer structures deform slowly at large angles. The bilayer structures are reversibly deformed, which shows excellent stability. The P(AAm-AAc-3-AAPBA) structure also shows good adhesive property in PVA solution. Our studies serve as a proof-of-concept that the bilayer structures of P(AAm-AAc-3-AAPBA)/PAAm are suitable for soft actuators, bionic flowers and manipulators. Due to slow deformation at some pH values, applications of the bilayer structures are restricted. For practical application, improvement in bilayer structures deformation at a lower pH and response to different stimuli can be investigated in our future study. Moreover, the mechanical property is important for applications. The compressive strength and bending modulus will be further investigated.

## 4. Conclusions

In this work, we presented a facile method to fabricate a bilayer hydrogel structure inspired by drosera by taking advantage of ionized/non-ionized states of 3-AAPBA and AAc and dynamic changes in intermolecular hydrogen bonds in different liquid mediums. The P(AAm-AAc-3-AAPBA) layer shows swelling/shrinking behaviors in response to different pH values of the liquid environment, and swelling/shrinking status of the PAAm layers is positively correlated with swelling/shrinking status of the P(AAm-AAc-3-AAPBA) layer, but the swelling/shrinking volume is much smaller than that of the P(AAm-AAc-3-AAPBA) layer. The effects of pH, 3-AAPBA content and glucose concentration on bending orientation, angle and rate were discussed. Controllable bending behaviors can be reversibly and repeatedly realized by cooperative swelling/shrinkage of the P(AAPBA-AAm-AAc)/PAAm bilayer structure. Except for their good bending property, the bilayer structures also show good bonding ability: they are able to fabricate complex hydrogel patterns. In addition, the bilayer hydrogel structures have been demonstrated as soft actuators, bionic flowers and bionic manipulators. The proposed facile method and new bilayer hydrogel system would be highly useful in design and fabrication of smart actuators with programmable and versatile properties.

## Figures and Tables

**Table 1:** The maximum bending angle versus pH.

pH	Maximum Bending Angle (°)	Time (min)
1	379 ± 1.41	360
2	372 ± 9.19	360
3	364 ± 18.38	360
4	436 ± 28.99	360
5	620 ± 28.28	360
6	725 ± 21.21	360
7	775 ± 35.36	360
8	730 ± 1.02	360
9	570 ± 22.63	360
10	510 ± 14.67	20
11	355 ± 0.71	20
12	−608 ± 2.77	20
13	−451 ± 1.27	20

## Data Availability

The data presented in this study are available on request from the corresponding author.

## Supplementary Materials

The following supporting information can be downloaded at: https://www.mdpi.com/article/10.3390/polym14204425/s1, Figure S1: The relationship between bending angle and time of different bilayer hydrogel placed in the solution at pH 1 (a), DI water (b) and at pH 13 (c); Figure S2: The relationship between bending angle and time of the P(AAm-AAc-3-AAPBA)/PAAm bilayer structures with different 3-AAPBA contents ((a) 0 wt%, (b) 2.5 wt%, (c) 5 wt%, (d) 7.5 wt%, (e) 10 wt%) at different pH.
