# Supplementary material for: Bioinspired High-Performance Bilayer, pH-Responsive Hydrogel with Superior Adhesive Property
Source: Polymers (Basel). 2022 Oct 19;14(20):4425. doi: 10.3390/polym14204425 (PMC9612346; doi:10.3390/polym14204425)
Supplement: Supplementary file 1 [file polymers-14-04425-s001.zip › polymers-1953267-supplementary.pdf]

Supporting information

Article

Bioinspired High-Performance Bilayer, pH-Responsive Hydrogel with Superior Adhesive Property

Shulan Jiang [1,*] and Li Xia [2]

[1] School of Mechanical Engineering and Electronic Information, China University of Geosciences (Wuhan),
Wuhan 430074, China
[2] Tribology Research Institute, School of Mechanical Engineering, Southwest Jiaotong University, Chengdu 610031, China
* Correspondence: jiangshulan@cug.edu.cn

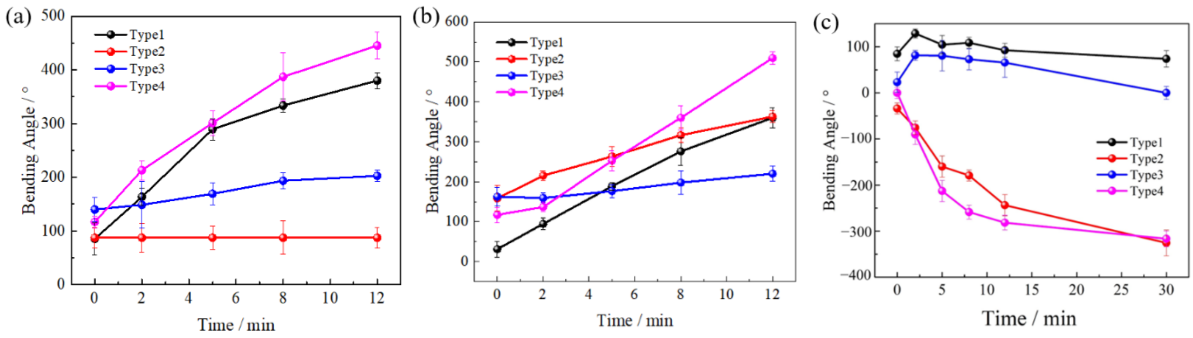

Figure S1. The relationship between bending angle and time of different bilayer hydrogel placed in the solution at pH 1(a), DI water (b) and at pH 13(c).

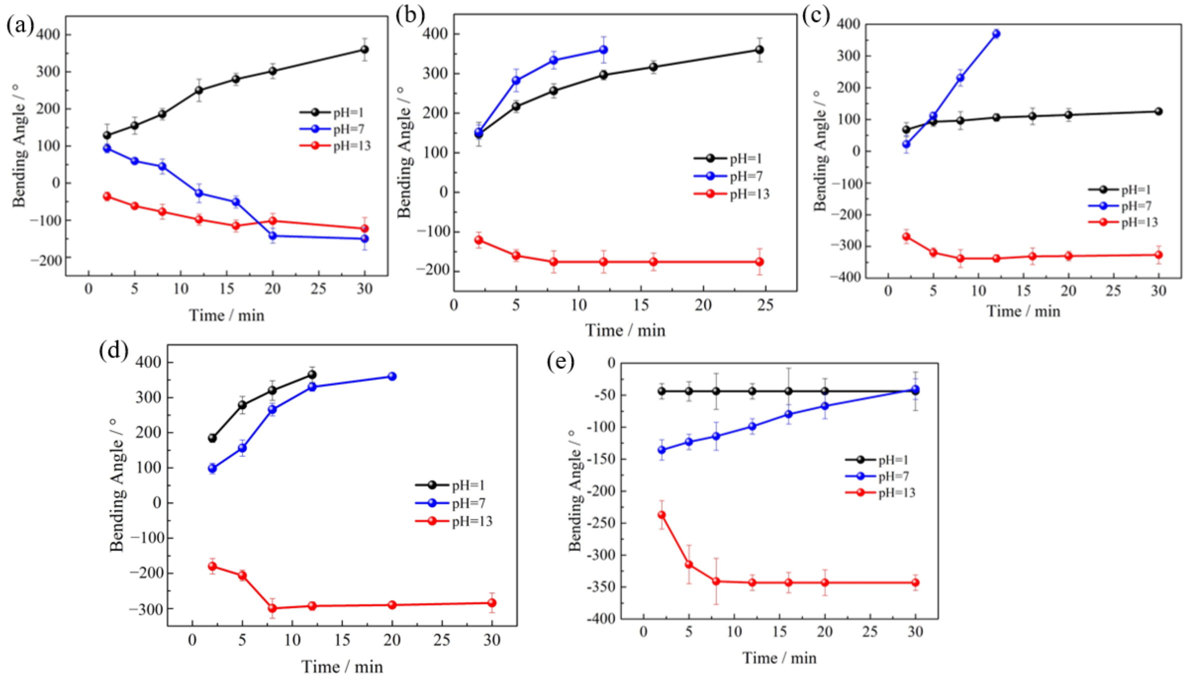

Figure S2. The relationship between bending angle and time of the P(AAm-AAc-3-AAPBA)/PAAm bilayer structures with different 3-AAPBA contents ((a) 0wt%, (b) 2.5wt%, (c) 5wt%, (d) 7.5wt%, (e) 10wt%) at different pH.
